# CD40 Receptor Knockout Protects against Microcystin-LR (MC-LR) Prolongation and Exacerbation of Dextran Sulfate Sodium (DSS)-Induced Colitis

**DOI:** 10.3390/biomedicines8060149

**Published:** 2020-06-02

**Authors:** Robin C. Su, Emily A. Warner, Joshua D. Breidenbach, Apurva Lad, Thomas M. Blomquist, Andrew L. Kleinhenz, Nikolai Modyanov, Deepak Malhotra, David J. Kennedy, Steven T. Haller

**Affiliations:** 1 Department of Medicine, The University of Toledo College of Medicine and Life Sciences, Toledo, OH 43614, USA; Robin.Su@rockets.utoledo.edu (R.C.S.); Emily.Warner2@rockets.utoledo.edu (E.A.W.); Joshua.Breidenbach@rockets.utoledo.edu (J.D.B.); Apurva.Lad@rockets.utoledo.edu (A.L.); Andrew.Kleinhenz@utoledo.edu (A.L.K.); Deepak.Malhotra@utoledo.edu (D.M.); 2 Department of Pathology, The University of Toledo College of Medicine and Life Sciences, Toledo, OH 43614, USA; Thomas.Blomquist@utoledo.edu; 3 Department of Physiology and Pharmacology, The University of Toledo College of Medicine and Life Sciences, Toledo, OH 43614, USA; Nikolai.Modyanov@utoledo.edu; 4 Department of Medical Microbiology and Immunology, The University of Toledo College of Medicine and Life Sciences, Toledo, OH 43614, USA

**Keywords:** CD40, inflammatory bowel disease, dextran sulfate sodium, colitis, microcystin, colon

## Abstract

Inflammatory Bowel Disease (IBD) is one of the most common gastrointestinal (GI) disorders around the world, and includes diagnoses such as Crohn’s disease and ulcerative colitis. The etiology of IBD is influenced by genetic and environmental factors. One environmental perturbagen that is not well studied within the intestines is microcystin-leucine arginine (MC-LR), which is a toxin produced by cyanobacteria in freshwater environments around the world. We recently reported that MC-LR has limited effects within the intestines of healthy mice, yet interestingly has significant toxicity within the intestines of mice with pre-existing colitis induced by dextran sulfate sodium (DSS). MC-LR was found to prolong DSS-induced weight loss, prolong DSS-induced bloody stools, exacerbate DSS-induced colonic shortening, exacerbate DSS-induced colonic ulceration, and exacerbate DSS-induced inflammatory cytokine upregulation. In addition, we previously reported a significant increase in expression of the pro-inflammatory receptor CD40 in the colons of these mice, along with downstream products of CD40 activation, including plasminogen activator inhibitor-1 (PAI-1) and monocyte chemoattractant protein-1 (MCP-1). In the current study, we demonstrate that knocking out CD40 attenuates the effects of MC-LR in mice with pre-existing colitis by decreasing the severity of weight loss, allowing a full recovery in bloody stools, preventing the exacerbation of colonic shortening, preventing the exacerbation of colonic ulceration, and preventing the upregulation of the pro-inflammatory and pro-fibrotic cytokines IL-1β, MCP-1, and PAI-1. We also demonstrate the promising efficacy of a CD40 receptor blocking peptide to ameliorate the effects of MC-LR exposure in a proof-of-concept study. Our findings suggest for the first time that MC-LR acts through a CD40-dependent mechanism to exacerbate colitis.

## 1. Introduction

Inflammatory bowel disease (IBD) is one of the most common gastrointestinal (GI) diseases around the world and is quickly increasing in prevalence, becoming a major global health concern [1]. Defined as a collection of diseases characterized by both acute and chronic inflammation, the term IBD is most familiarly known to include disorders such as Crohn’s disease and ulcerative colitis [2,3]. In the USA alone, IBD has grown in prevalence from 1.8 million affected individuals in 1999 to 3.1 million in 2015 [4].

IBD is a disease with a multifactorial etiology that can include genetic components, environmental influences, and factors that dysregulate the immune system and GI microbiota [5]. While understanding the genetic etiologies of IBD is very important, it is equally, if not more important, to investigate and elucidate environmental perturbagens, as exposure to these can often be modulated. Many of these environmental factors have been well established and include smoking, diet, antibiotic and NSAID use, and microorganism infections [5].

One of the environmental factors that remains to be considered in IBD populations is microcystin. Microcystins are toxins produced by cyanobacteria, or blue-green algae, in freshwater environments around the world, with harmful algal blooms (HABs) increasing in frequency and severity globally every year [6,7]. In fact, 40% of lakes and reservoirs in Europe, Asia, and America have favorable environments for HABs [8]. Of the various forms of microcystins, microcystin-leucine arginine (MC-LR) is one of the most frequently produced and one of the most toxic forms [9]. MC-LR has been most notorious for its severe liver toxicity, but has also been found to have other potential toxic effects within the reproductive system, heart, and kidneys, and has even been categorized as a potential human carcinogen [9,10,11,12,13,14,15,16]. MC-LR’s effects in humans have recently been extensively reviewed [17]. While cases of MC-LR toxicity in humans are well documented around the world, one of the most notable cases occurred in Brazil in 1996 [18,19]. At a clinic in Brazil, 100 of 130 dialysis patients were diagnosed with acute liver failure and 52 of those patients died soon after due to a syndrome called “Caruaru Syndrome” [18,19,20]. It was recognized later that the clinic had been receiving untreated water, with the documented deaths being attributed to intravenous exposure to microcystins [18,19,20].

While MC-LR is extensively studied within the liver, its effects within other organ systems is not as extensively characterized. The effects of MC-LR also need to be better understood in the setting of pre-existing disease conditions. In fact, we have recently reported its severe toxicity in the liver at low, chronic doses in the setting of pre-existing non-alcoholic fatty liver disease (NAFLD) [21,22]. This has raised the concern that populations with pre-existing disease conditions should be urgently studied given their potentially increased susceptibility to MC-LR toxicity.

We have recently reported the first investigation studying the effects of MC-LR within the intestines in both healthy settings and in the setting of pre-existing IBD, as MC-LR is one of the several potential environmental factors that have not been studied in the setting of IBD [23]. We observed that MC-LR has limited effects within the intestines of healthy C57BL/6 mice, yet had significant toxicity within the intestines of C57BL/6 mice with pre-existing colitis as induced by dextran sulfate sodium (DSS) [23]. One of the most notable findings we observed from our previous study was that the CD40 receptor was significantly upregulated in the colons of mice with pre-existing colitis that were exposed to MC-LR [23]. Furthermore, we found that downstream products of CD40 activation, monocyte chemoattractant protein-1 (MCP-1) and plasminogen activator inhibitor-1 (PAI-1), were also upregulated in the same group [23]. Because CD40 is a prominent immunomodulatory receptor found on antigen-presenting immune cells and has recently been extensively linked to IBD pathogenesis [24,25], we hypothesized that CD40 might play a key role in mediating MC-LR induced exacerbation of IBD. In order to address this hypothesis, the present study investigates the effects of MC-LR within the intestines or CD40 receptor knockout (CD40KO) mice with and without pre-existing colitis as compared with wild type (WT) C57 mice, with the hypothesis that knocking out CD40 would attenuate the effects of MC-LR in mice with pre-existing colitis. In addition, we further confirmed the role of CD40 by utilizing a CD40 receptor blocking peptide (CD40 peptide) in WT mice in a proof-of-concept study. Together, we believe our novel findings through the use of CD40KO mice and a CD40 blocking peptide in WT mice elucidate the important role of CD40 in MC-LR exacerbation of colitis and its potential as a therapeutic target in populations with pre-existing that are more vulnerable and susceptible to MC-LR toxicity.

## 2. Results

### 2.1. Body Weight and Survival

**Figure 1:**
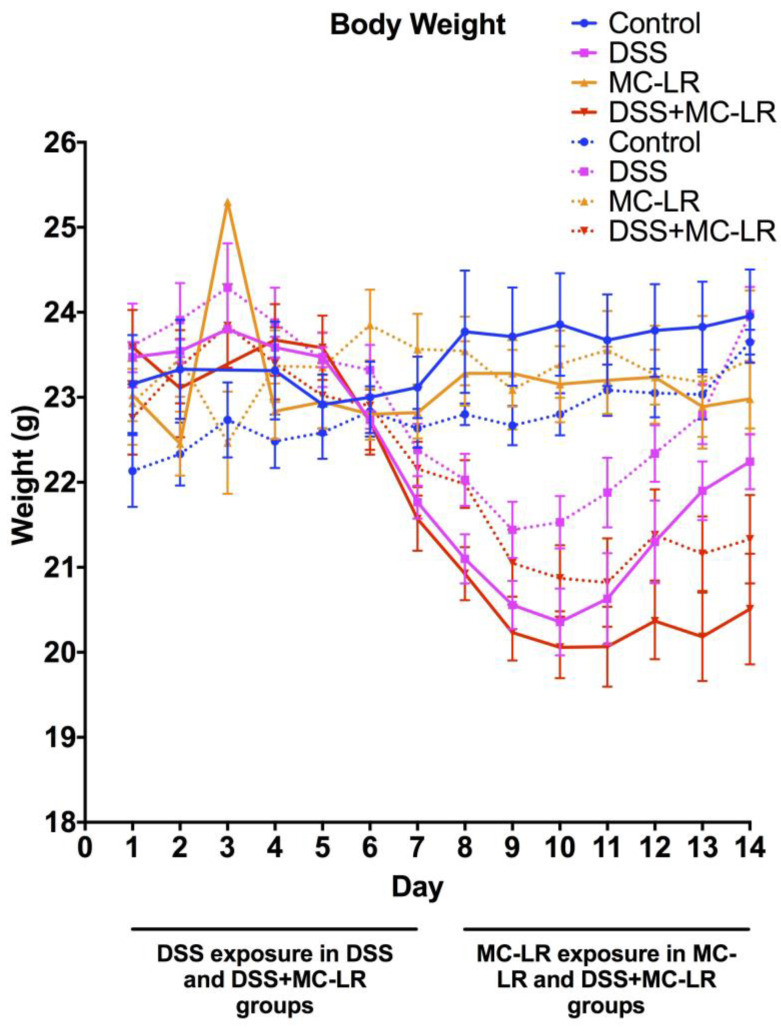
Mouse body weights measured daily throughout the 14-day study. Data presented indicate the mean ± SEM (*n* = 6–11 mice per group). Solid lines indicate wild type (WT) mice. Dotted lines indicate CD40KO mice.

Overall, CD40KO mice in all four groups (control, DSS, MC-LR, and DSS + MC-LR) showed similar patterns of body weight to the four groups of WT mice. Mice in the MC-LR group showed no significant differences in body weight compared with control mice throughout the 14-day study (*p* = 0.99) (Figure 1). Mice in the DSS group and in the DSS + MC-LR group showed decreases in body weight. DSS mice showed full recovery in body weight following removal of DSS during the second week of the study until there were no differences in body weight as compared with the control and MC-LR groups (*p* = 0.98). Mice in the DSS + MC-LR group did not show recovery in body weight but had prolonged loss of body weight until the end of the study, where their body weights remained significantly lower than the control, MC-LR, and DSS groups (*p* < 0.05). While CD40KO mice had similar pattern to WT mice, the loss in body weight of CD40KO mice in DSS and DSS + MC-LR groups appeared to be less severe than WT mice in the same groups; however, these trends were not statistically significant (*p* = 0.38 and *p* = 0.94, respectively). It is also important to note that there was one mortality in the WT DSS + MC-LR group before the conclusion of the study. No mortality was observed in the CD40KO DSS + MC-LR group.

### 2.2. Stool Grading

**Figure 2:**
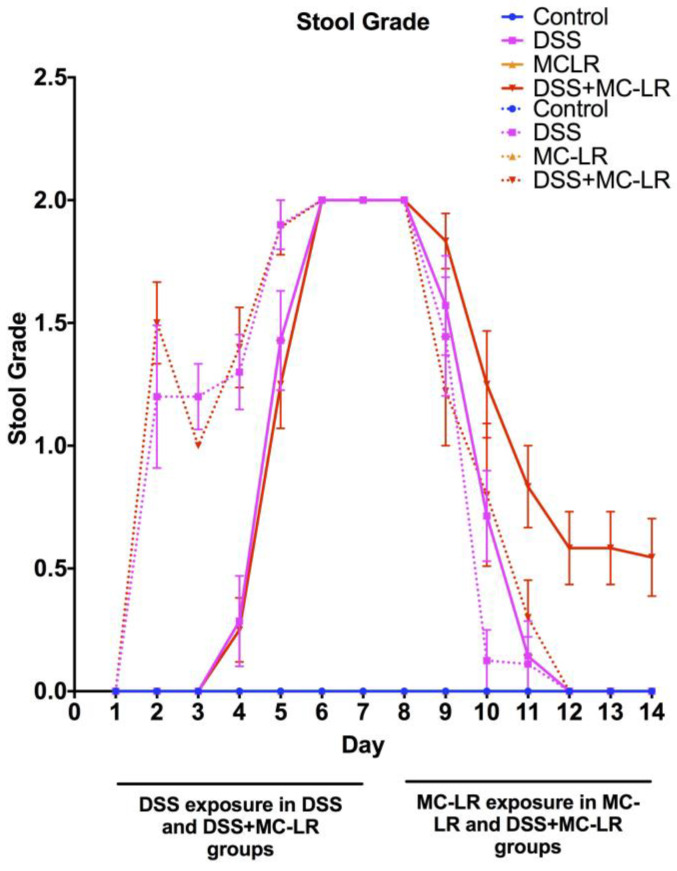
Daily stool grading throughout the 14-day study; 0 = no occult or gross blood, 1 = occult blood present, and 2 = gross blood present. Data presented indicate the mean ± SEM (*n* = 6–11 mice per group). Solid lines indicate WT mice. Dotted lines indicate CD40KO mice.

In both WT and CD40KO mice, control and MC-LR groups showed no occult or gross blood in their stool throughout the 14-day study (Figure 2). WT and CD40KO mice in the DSS group showed increased occult and gross blood in their stool with increased duration of DSS exposure. There was subsequently full recovery in their stool following DSS removal, until there was no longer any detectable blood within their stool. WT mice in the DSS + MC-LR group experienced increased occult and gross blood in their stool with increased duration of DSS exposure but did not show full recovery in their stool following DSS removal, with prolonged occult blood still being present by the end of the study (*p* < 0.05). While CD40KO mice in the DSS + MC-LR group also experienced increased occult and gross blood in their stool with increased duration of DSS exposure, they interestingly showed complete recovery in their stool following DSS removal, until there was no longer any detectable blood within their stool.

### 2.3. Colon Length

**Figure 3:**
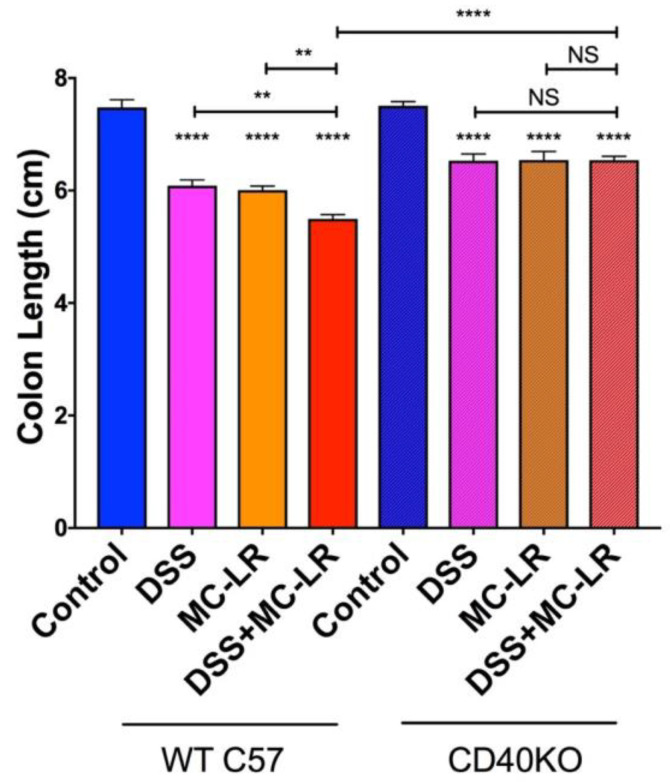
Effect of dextran sulfate sodium (DSS) and microcystin-leucine arginine (MC-LR) on colon length. Data presented indicate the mean ± SEM (*n* = 6–11 mice per group). ** *p* < 0.01 and **** *p* < 0.0001.

Colons were measured from the distal end to the colon-cecum junction. Colons of WT and CD40KO mice in the DSS, MC-LR, and DSS + MC-LR groups showed significant decreases in length as compared with the control groups (Figure 3). Overall colonic shortening in the CD40KO mice was significantly less severe than in the WT mice. Additionally, WT mice in the DSS + MC-LR showed further colonic shortening as compared with DSS and MC-LR groups, whereas no such exacerbation of colonic shortening was seen in the CD40KO mice in the DSS + MC-LR group compared with DSS and MC-LR groups.

### 2.4. Histopathology

**Figure 4:**
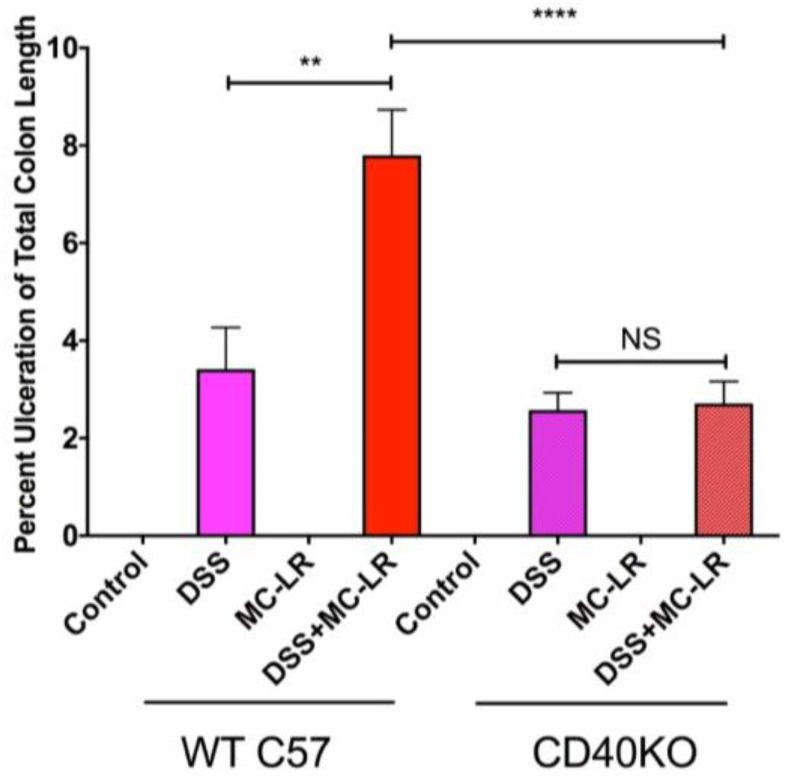
Effect of DSS and MC-LR on colonic ulceration. Total ulcer length throughout the colon was normalized to total colon length. Data presented indicate the mean ± SEM (*n* = 6–11 mice per group). ** *p* < 0.01 and **** *p* < 0.0001.

Histopathological analysis of hematoxylin and eosin (H&E) stained colon sections revealed that DSS exposure and DSS + MC-LR exposure led to segmental regions of ulceration, crypt abscesses, marked acute inflammatory cell infiltration, and early architectural distortion with gland branching and budding (i.e., early chronic changes), as compared with the normal colonic tissue of the control group. Measurement of the length of ulcerated mucosa to total colon length demonstrates significantly greater colonic ulceration in the WT DSS + MC-LR group as compared with WT DSS mice (Figure 4). Conversely, no differences in colonic ulceration were observed in the CD40KO DSS + MC-LR group as compared with CD40KO DSS mice, and the percent ulceration in these two CD40KO groups did not differ from that of WT DSS mice but was significantly lower than WT DSS + MC-LR mice (Figure 4).

**Figure 5:**
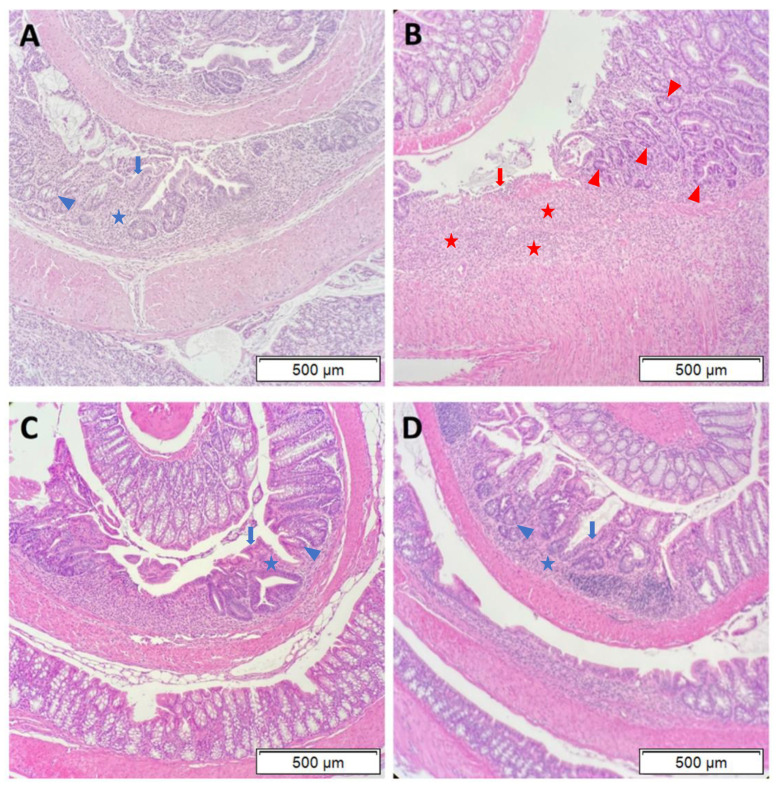
Representative hematoxylin and eosin (H&E) stained sections of colon histopathology. (**A**) WT with DSS exposure. Patchy areas of ulceration were noted, with evidence of re-epithelialization, indicating wound healing processes (blue arrows). In addition to these areas of ulceration, there are moderate areas of inflammatory infiltration (blue stars). Adjacent to these areas, gland architecture is modestly distorted with reactive atypia (nuclear enlargement, hyperchromasia, and prominent nucleoli) (blue triangular arrowheads). (**B**) WT with DSS + MC-LR exposure. Large areas of ulceration were noted, with an absence of re-epithelialization, indicating an inhibition of wound healing processes (red arrows). In addition to these large areas of ulceration, there is heavy inflammatory infiltration with fibrin deposition (red stars). Adjacent to these areas, gland architecture is severely distorted with aggressive reactive atypia in the gland architecture (red triangular arrowheads). (**C**) CD40KO with DSS exposure. Patchy areas of ulceration were noted, with evidence of re-epithelialization, indicating wound healing processes (blue arrows). In addition to these areas of ulceration, there are moderate areas of inflammatory infiltration (blue stars). Adjacent to these areas, gland architecture is modestly distorted with reactive atypia (blue trianglular arrowheads). (**D**) CD40KO with DSS + MC-LR exposure. Patchy areas of ulceration were noted, with evidence of re-epithelialization, indicating wound healing processes (blue arrows). In addition to these areas of ulceration, there are moderate areas of inflammatory infiltration (blue stars). Adjacent to these areas, gland architecture is modestly distorted with reactive atypia (blue trianglular arrowheads). All images were taken at 10× objective. Scale bars indicate 500 μm.

The marked histopathology seen in WT and CD40KO mice with DSS and DSS + MC-LR exposure was further characterized. In the WT DSS mice (Figure 5A), there was evidence of patchy ulceration, with a moderate degree of mixed acute and chronic inflammatory infiltrates (blue stars). These areas of focal ulceration had evidence of focal re-epithelialization (blue arrows). Adjacent to these areas of ulceration, the gland architecture is modestly distorted with reactive atypia (nuclear enlargement, hyperchromasia, and prominent nucleoli) (blue trianglular arrowheads). These findings of inflammation, architectural disruption, and nuclear changes are consistent with what has been previously identified with DSS exposure in mice [26,27,28]. In the WT DSS + MC-LR mice (Figure 5B), there were much larger areas of ulceration with heavy mixed inflammatory cell infiltrates and overlying fibrin deposition (red stars). In contrast to WT DSS mice, WT DSS + MC-LR mice exhibited minimal re-epithelialization of the areas of ulceration (consistent with an inhibited wound healing process) (red arrows). The adjacent mucosa was also hyperplastic with a more exuberant reactive atypia in the gland architecture (red triangular arrowheads). In CD40KO DSS mice (Figure 5C), as is similarly observed in CD40KO DSS + MC-LR mice (Figure 5D), there is a moderate inflammatory infiltrative process (similar to WT DSS mice) (blue stars). Moreover, CD40 DSS and DSS + MC-LR both showed re-epithelialization of ulcers (blue arrows) and only the modest gland reactive atypia (blue triangular arrowheads) seen in WT DSS mice.

### 2.5. Gene Expression in the Colon

**Figure 6:**
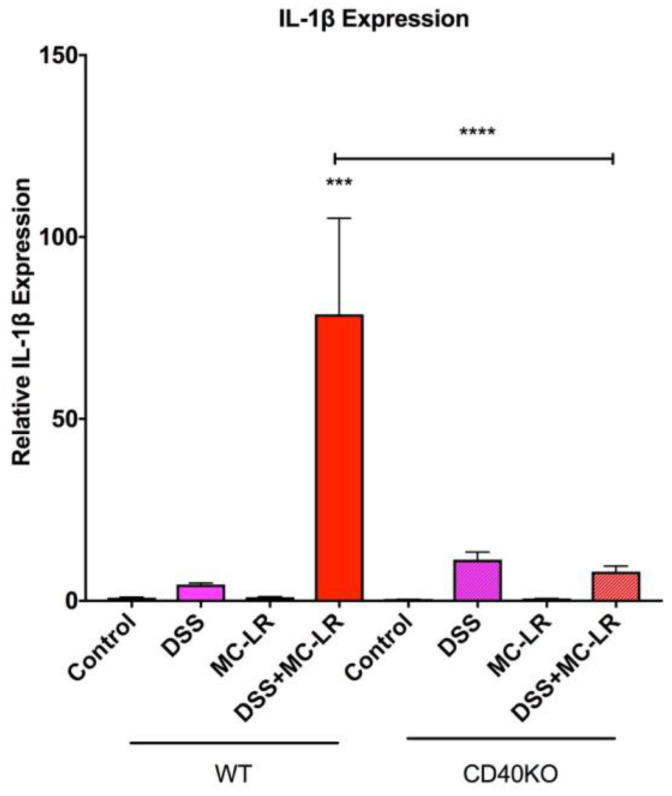
RT-qPCR analysis of pro-inflammatory cytokine IL-1β. Data presented indicate the mean ± SEM (*n* = 6–11 mice per group). *** *p* < 0.001 and **** *p* < 0.0001.

As seen in Figure 6, the qPCR analysis demonstrated that the mRNA levels of the pro-inflammatory cytokine IL-1β was significantly upregulated only in the WT DSS + MC-LR group as compared with the WT DSS group. This IL-1β upregulation was not observed in the CD40KO DSS + MC-LR group and IL-1β was significantly lower in the CD40KO DSS + MC-LR group as compared with the WT DSS + MC-LR group, demonstrating that knocking out CD40 prevents the subsequent upregulation of the pro-inflammatory marker IL-1β.

**Figure 7:**
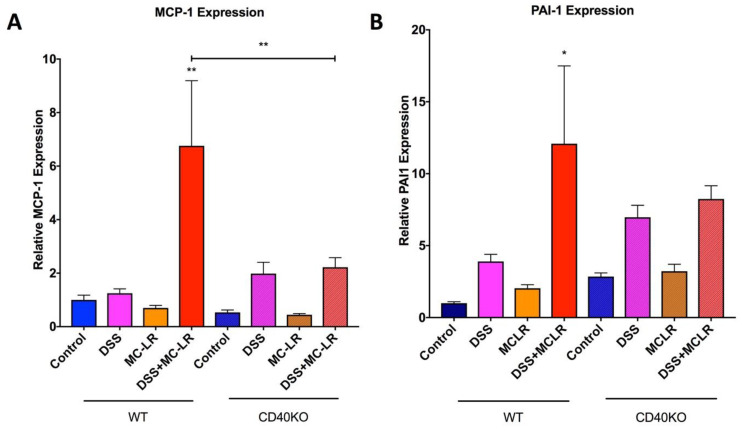
RT-qPCR analysis of pro-inflammatory and pro-fibrotic cytokines and downstream products of CD40 activation. (**A**) Relative MCP-1 expression. MCP-1 expression was significantly elevated with DSS + MC-LR exposure in WT mice, however, MCP-1 was not significantly elevated with DSS + MC-LR exposure in CD40KO mice. (**B**) Relative PAI-1 expression. Relative PAI-1 expression was significantly elevated with DSS + MC-LR exposure in WT mice, however, PAI-1 was not significantly elevated with DSS + MC-LR exposure in CD40KO mice. Data presented indicate the mean ± SEM (*n* = 6–11 mice per group). * *p* < 0.05 and ** *p* < 0.01.

qPCR analysis for downstream products of CD40 activation, MCP-1 and PAI-1, reveal a significant increase in MCP-1 in the WT DSS + MC-LR group as compared with the WT control group (Figure 7A). MCP-1 was not upregulated in the CD40KO DSS + MC-LR group and there is a significantly lower MCP-1 level in the CD40KO DSS + MC-LR group as compared with WT DSS + MC-LR group, demonstrating that knocking out CD40 prevents the subsequent production of the pro-inflammatory cytokine MCP-1 even in the setting of MC-LR exposure in pre-existing colitis (Figure 7A). Similarly, PAI-1 was found to be significantly upregulated only in the WT DSS + MC-LR group as compared with the WT control group (Figure 7B). PAI-1 was not upregulated in the CD40KO DSS + MC-LR group, demonstrating that knocking out CD40 prevents the subsequent production of the pro-fibrotic cytokine PAI-1 (Figure 7B).

### 2.6. Proof-of-Concept Study with CD40 Receptor Blocking Peptide

**Figure 8:**
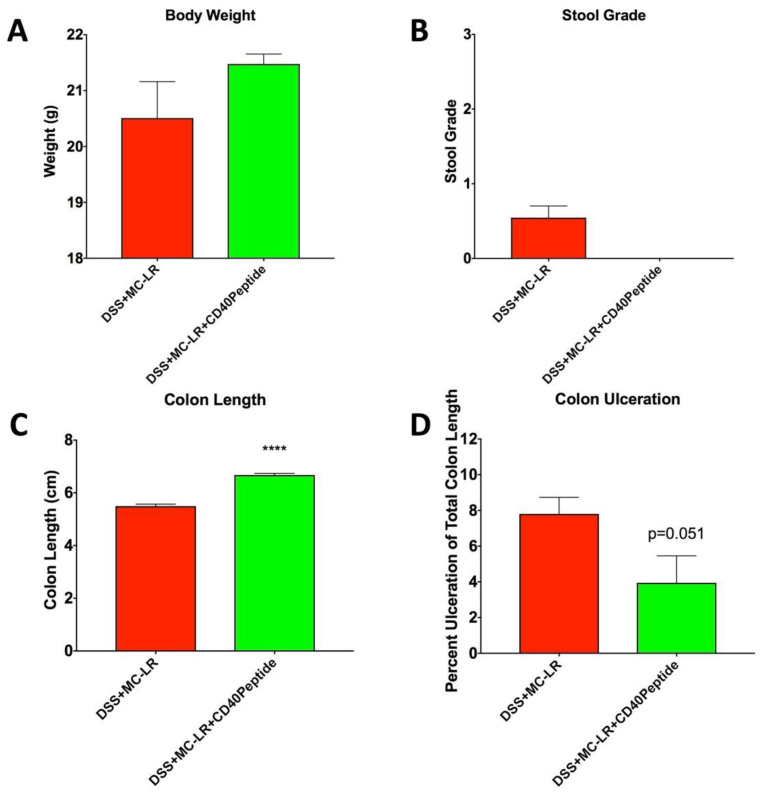
CD40 blocking peptide treatment in mice with pre-existing colitis and MC-LR exposure. (**A**) Body weight. Endpoint bodyweight of mice with DSS + MC-LR + CD40 peptide exposure was greater, although not significantly, than mice with DSS + MC-LR exposure with CD40 peptide treatment, showing a trend in bodyweight recovery with CD40 peptide treatment. (**B**) Stool grade. At the endpoint of the study, mice that received DSS + MC-LR exposure had blood that persisted in their stool. However, with CD40 treatment on top of DSS + MC-LR exposure, mice had full recovery in their stool, with no blood present. (**C**) Colon length. Colon length was significantly longer with CD40 peptide treatment versus without treatment in mice given DSS + MC-LR exposure. (**D**) Colon ulceration. Percent ulceration of total colon length was decreased with CD40 peptide treatment than without treatment in mice given DSS + MC-LR exposure. Data presented indicate the mean ± SEM (*n* = 4–11 mice per group). **** *p* < 0.0001.

To further demonstrate the importance of CD40 in MC-LR’s mechanism of toxicity in the setting of pre-existing colitis, we utilized a CD40 receptor blocking peptide within WT mice in a proof-of-concept study. Interestingly, treatment with the CD40 peptide resulted in a reduction in MC-LR effects in the setting of pre-existing colitis that paralleled that which was seen in the CD40KO mice. While WT DSS + MC-LR + CD40 peptide mice experienced the DSS-induced decrease in body weight, the decrease appeared less severe than in the WT DSS + MC-LR mice without CD40 peptide treatment, as was also seen in the CD40KO mice (Figure 8A). This trend, however, was not statistically significant (*p* = 0.40). Similar to the CD40KO mice, WT DSS + MC-LR + CD40 peptide mice developed DSS-induced bloody stools. However, the WT DSS + MC-LR + CD40 peptide mice demonstrated full recovery by the end of the study with the removal of DSS (Figure 8B). This, again is compared to the persistent bloody stools observed in the WT DSS + MC-LR mice without CD40 peptide treatment. Similar to CD40KO mice, CD40 peptide treatment prevented MC-LR exacerbation of DSS-induced colonic shortening, with colon length being significantly greater with peptide treatment than without (Figure 8C). Similar to CD40KO mice, CD40 peptide treatment prevented MC-LR exacerbation of DSS-induced colonic ulceration (*p* = 0.051) (Figure 8D).

**Figure 9:**
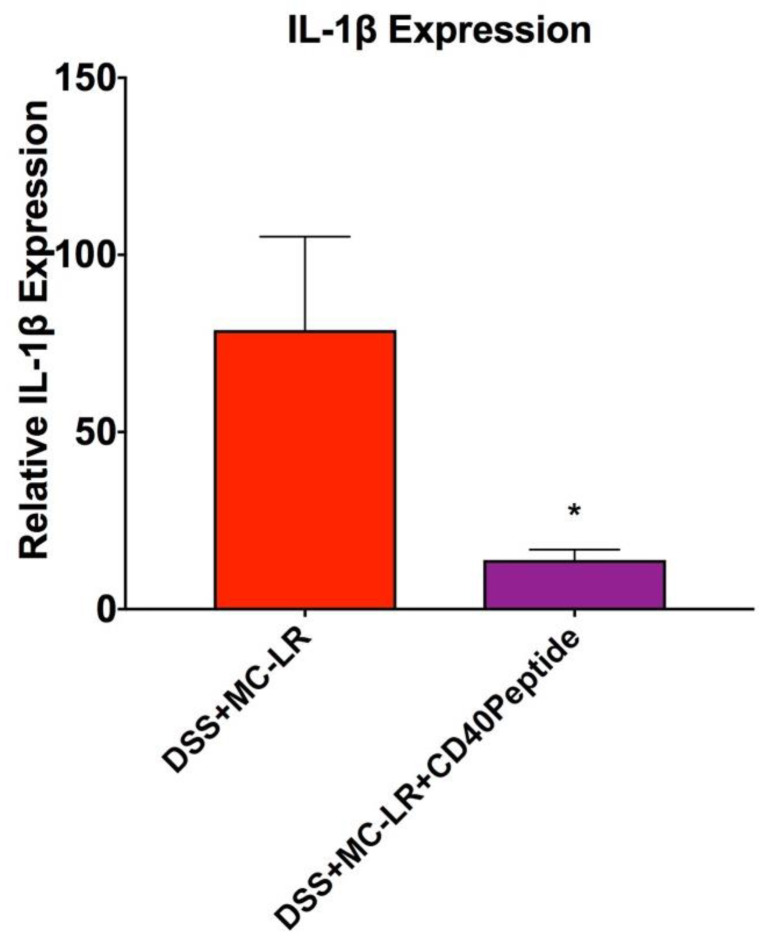
RT-qPCR analysis of pro-inflammatory cytokine IL-1β. Data presented indicate the mean ± SEM (*n* = 4–11 mice per group). * *p* < 0.05

qPCR analysis also confirmed the effectiveness of CD40 peptide treatment in mice with pre-existing colitis and MC-LR exposure. Similar to what was seen in the CD40 KO mice, treatment of DSS + MC-LR mice with CD40 peptide resulted in significant decreases in expression of the pro-inflammatory cytokine IL-1β in the colon tissue (Figure 9). Additionally, colonic expression of the pro-inflammatory and pro-fibrotic cytokines MCP-1 and PAI-1 were decreased in the CD40 peptide treated group; however, this was not statistically significant (for MCP-1: 6.8 ± 2.4 DSS + MC-LR vs 2.4 ± 1.0 DSS + MC-LR + CD40 blocking peptide, *p* = 0.30; For PAI-1: 12.1 ± 5.4 DSS + MC-LR vs 3.4 ± 0.6 DSS + MC-LR + CD40 blocking peptide, *p* = 0.35).

## 3. Discussion

This study is the first to examine the effects of the CD40 receptor in modulating MC-LR toxicity within the intestines. We have previously shown that MC-LR has limited effects within the intestines of healthy WT C57BL/6 mice, yet has severe toxicity in the intestines of mice with pre-existing colitis induced by DSS [23]. In WT mice, MC-LR is found to prolong DSS-induced weight loss, prolong the presence of DSS-induced blood within stool, exacerbate DSS-induced colonic shortening, exacerbate DSS-induced ulceration of colonic mucosa, and exacerbate DSS-induced inflammatory cytokine upregulation in mice with DSS-induced colitis as compared with mice with colitis and no MC-LR exposure [23]. We also observed the upregulation of the CD40 receptor as well as the downstream products of CD40 activation, MCP-1, and PAI-1, within the colons of mice with DSS-induced colitis and subsequent MC-LR exposure. In the current study, we observed that knocking out CD40 ameliorates the effects of MC-LR within the intestines of mice with DSS-induced colitis and also show that treatment with a CD40 blocking peptide mirrors the effects seen with knocking out CD40.

When examining body weight within the CD40KO mice, we observed that mice exposed to MC-LR do not differ in body weight as compared with controls, mice with DSS-induced colitis experience a decrease in body weight and a full recovery in body weight upon removal of DSS, and mice with colitis and MC-LR experience a prolonged decrease in body weight without recovery. This is a similar pattern as what is seen in the WT mice; however, it is important to note that the decrease in body weight appears to be less severe in the CD40KO mice as compared with the WT mice, though this is not statistically significant. While DSS + MC-LR exposure caused prolonged bloody stools without recovery following DSS removal in WT mice, knocking out CD40 led to full recovery of bloody stools following DSS removal. In addition, no exacerbation of DSS-induced colonic shortening and no exacerbation of DSS-induced colonic ulceration was observed in CD40KO mice with colitis and MC-LR exposure as compared to mice with colitis alone. We also observed that, while the pro-inflammatory and pro-fibrotic cytokines IL-1β, MCP-1, and PAI-1 were upregulated in the WT DSS + MC-LR group, knocking out CD40 prevented their upregulation upon MC-LR exposure in the setting of DSS-induced colitis. It is also important to note that mortality (1) was observed in the WT group with colitis and MC-LR exposure, indicating heightened severity of disease, whereas no mortality was observed in the CD40KO group with colitis and MC-LR exposure. We also demonstrated that treatment with a CD40 receptor blocking peptide also decreases the severity of weight loss, allowed full recovery of bloody stools, decreased colonic shortening, decreased colonic ulceration, and decreased the levels of the pro-inflammatory markers IL-1β in WT DSS + MC-LR mice as compared with no CD40 peptide treatment. Collectively, these findings suggest that the CD40 receptor plays a role in mediating the toxic effects of MC-LR in the setting of pre-existing colitis.

CD40 is a receptor belonging to the TNF superfamily and is ubiquitously expressed on immune cells, such as B cells, macrophages, and dendritic cells, as well as nonimmune cells, such as platelets, endothelial cells, epithelial cells, and mesenchymal cells [25,29,30]. The CD40 ligand (CD40L) is also expressed on a variety of cells, though it is primarily expressed on activated CD4+ T cells and in the circulation as an active, soluble form (sCD40L) [31]. The CD40-CD40L binding interaction leads to numerous different intracellular cascades, depending on the cell type and physiological environment, activating a broad spectrum of kinases, such as ERK, JNK, and MAPK, and further activating downstream transcription factors, such as NFkB, NFAT, and AP-1 [32,33,34].

The CD40-CD40L interaction is crucial for many important immune and inflammatory processes. CD40 activation is a key driver in B cell activation and drives immunoglobulin isotype switching [35,36,37]. In fact, genetic mutation of CD40L leads to a compromising X-linked disease called hyper-IgM syndrome, in which patients’ B cells are unable to produce other subtypes of immunoglobulins aside from the default IgM due to the absence of CD40 activation by normal CD40L [35,38,39]. In addition to driving adaptive immune responses, CD40 also drives innate immune responses through dendritic cells and monocytes/macrophages [40]. CD40 dependent activation of macrophages leads to important inflammatory and pro-fibrotic cytokine production, including IL-1β, MCP-1, and PAI-1 that perpetuate innate immune responses [40,41,42,43,44].

Systemically, prolonged or accentuated CD40-dependent responses can lead to disease progression. For example, prolonged CD40 activation in the renal proximal tubular epithelial cells can lead to chronic inflammatory kidney diseases [45]. In fact, our previous investigations have shown CD40 to be important in the pathogenesis of renal fibrosis and inflammation in the setting of hypertension and atherosclerotic renal artery stenosis, and actually predictive of renal function in patients with chronic kidney disease [43,46,47,48,49].

Interestingly, CD40 has recently be found to be a key contributor to the pathogenesis of IBD. Immunohistochemical staining for CD40 has revealed that, while colonic tissue of healthy patients exhibits weak overall staining, colonic tissue of IBD patients exhibits strong staining of B, mononuclear, endothelial, and mesenchymal cells, and its overexpression level has been shown to directly correlate to disease severity [50,51,52,53]. This enhanced immunohistochemical staining in IBD patient colons is true for CD40L as well [53,54]. In addition, while CD40 is not normally expressed on healthy intestinal epithelial cells, recent findings reveal that CD40 is actually expressed on intestinal epithelial cells in inflamed colon regions of patients with IBD [55]. Preliminary studies have found that these upregulations in CD40 and CD40L in IBD inflamed colons, on both immune cells and intestinal epithelial cells, drive pathological inflammatory cytokine upregulation, contributing to the enhanced inflammatory state in IBD [52,53,54,56].

Our previous study found that MC-LR further enhances the upregulation of CD40 in the setting of pre-existing colitis, along with upregulation of downstream products of CD40 activation, PAI-1 and MCP-1. While CD40 has been shown to be a key driver in IBD pathogenesis, our findings suggested that MC-LR exacerbates IBD through CD40. Our current, novel findings further suggest that knocking out CD40 effectively attenuates some of the effects of MC-LR in the setting of pre-existing IBD and treatment with a CD40 receptor blocking peptide mirrors the effects seen with knocking out CD40. Future studies will help elucidate potential preventative and therapeutic measures surrounding CD40 that can be used to combat MC-LR GI toxicity in more vulnerable and susceptible populations, such as those with pre-existing colitis.

## 4. Materials and Methods

### 4.1. Mice and Experimental Design

**Figure 10:**
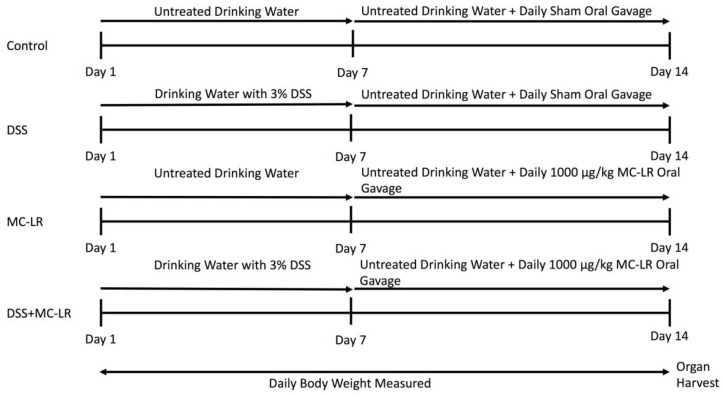
Experimental design evaluating the effects of DSS exposure, MC-LR exposure, and DSS + MC-LR exposure in the colons of C57BL/6 mice and CD40KO mice.

Animal experimentation was conducted in accordance with the National Institutes of Health (NIH) Guide for the Care and Use of Laboratory Animals. Protocols for animal experimentation were approved by The University of Toledo Institutional Animal Care and Use Committee (IACUC protocol #108663, approval date 9 February 2016). All mice were housed in a specific pathogen-free facility. Mice were maintained at standard conditions of 23 ± 1 °C under a 12-h light cycle and were allowed to eat a normal chow diet ad libitum. Male C57BL/6 mice and B6.129P2-Cd40^tm1Kik^/J mice were purchased from The Jackson Laboratory (Jackson Laboratory, Bar Harbor, ME, USA) at seven weeks of age. Upon arrival to the animal facility, the mice were assigned randomly to one of four groups: (a) Water only (control), (b) DSS followed by water (DSS), (c) water followed by MC-LR (MC-LR), and (d) DSS followed by MC-LR (DSS + MC-LR). Within the WT strain, the control water group consisted of seven mice, the DSS group consisted of seven mice, the MC-LR group consisted of eleven mice, and the DSS + MC-LR group consisted of twelve mice. Within the CD40KO strain, the control water group consisted of six mice, the DSS group consisted of ten mice, the MC-LR group consisted of nine mice, and the DSS + MC-LR group consisted of ten mice. All mice were allowed to acclimatize to their new environment until eight weeks of age. At eight weeks of age, mice were initiated into the study. The experimental design followed was as previously outlined [23]. The experimental plan and timeline are also summarized in Figure 10. Briefly, control mice were allowed to drink water ad libitum for the entire 14-day study. Control mice were also orally gavaged with water on a daily basis from day 8 to 14 as a sham procedure. Mice in the DSS group were exposed to 3% DSS (MP Biomedical, Solon, OH, USA, Item No. 0216011080) in drinking water, which they were allowed to drink ad libitum from days 1-7 according to established protocols [28]. Between day 8 and 14, these mice were allowed to drink untreated water ad libitum, while also being given water by daily sham oral gavage. Mice in the MC-LR group were allowed to drink water ad libitum for the entire 14-day study. From day 8 to 14, these mice were orally gavaged 1000 μg/kg MC-LR (Cayman Chemical, Ann Arbor, MI, USA, item no. 10007188) daily. The DSS + MC-LR group of mice were allowed to drink 3% DSS water ad libitum from days 1–7 and then were allowed to drink untreated water ad libitum from day 8 to 14. During days 8–14, these mice were orally gavaged 1000 μg/kg MC-LR daily. Weight of each mouse was measured daily. Stool was evaluated daily for the presence of occult and gross blood. Occult blood was measured using the Beckman Coulter Hemoccult Single Slides kit (Med Plus Physician Supplies, Edison, New Jersey, USA, Catalog #BC-60151). All mice were euthanized on day 15 and organs were harvested and weighed immediately following euthanasia. Colons (with cecum still intact) were measured adjacent to a standard ruler and photographs were taken. Following removal of the cecum and thorough washing of the colon with PBS (Fisher Scientific, Hampton, NH, USA, Catalog # SH3025601), sections of distal and proximal colon were taken from each sample, flash frozen in liquid nitrogen, and subsequently stored at −80 °C for future qPCR analysis.

We also completed a proof-of-concept study to observe the effects of treatment with a CD40 receptor blocking peptide designed with the sequence described by Vaitaitis et al. [57] (Ohio Peptide LLC., Powell, OH, USA). Four C57BL/6 mice were used and followed the same experimental plan outlined for WT DSS + MC-LR mice. In addition to this, these mice received 200 μL CD40 peptide (2 mg/kg body weight) by retro-orbital injection with mice under inhaled anesthesia once daily from day 10 to 14.

### 4.2. Histology

During organ harvesting following euthanasia, colonic tissue from all mice were cut longitudinally, wrapped around a rigid holder, placed in tissue cassettes, and fixed in 10% neutral buffered formalin for 24 h. Following 24 h in formalin, cassettes were transferred to 70% ethanol. Formalin-fixed tissues were then processed and embedded in paraffin (FFPE). Five (5) micron tissue sections were cut and placed on glass slides and stained with hematoxylin and eosin (H&E). Images of histology slides were taken using an Olympus CKX53 microscope and Olympus CellSens software (Standard 1.15, Olympus, Center Valley, PA, USA). Histopathological analysis was performed using H&E stained sections as assessed by a board certified Pathologist (T.M.B.) who was blinded to the group assignments. Colonic ulceration was quantified using the Olympus CellSens software by measuring the total length of the ulcerated colon and normalizing to the total length of the colon to give percent ulceration of total colon length.

### 4.3. RNA Extraction and RT-qPCR Method

RNA extraction, cDNA preparation, and RT-qPCR were all performed utilizing the QIAGEN (Qiagen, Germantown, MD, USA) automated liquid handling workflow system (QIAcube HT and QIAgility). RNA from distal colonic tissue (flash-frozen and stored at −80 °C) was isolated utilizing the QIAzol/chloroform extraction methodology. RNA was purified using the lithium chloride method as previously published [58]. Approximately 500 ng of extracted RNA was used to synthesize cDNA (QIAGEN’s RT2 First Strand Kit). RT-qPCR was performed utilizing QIAGEN’s Rotor-Gene Q thermo-cycler. The calculation of gene expression was conducted by comparing the relative change in cycle threshold value (ΔCt). Fold change in expression was calculated using the 2-ΔΔCt equation as previously described [59]. The IL-1β (Mm00434228_m1), MCP-1 (Mm00441242_m1), and PAI-1 (Mm00435858_m1) Taqman primers were used and obtained from Thermo Fisher Scientific (Thermo Fisher Scientific, Waltham, MA, USA). 18s rRNA from Thermo Fisher Scientific was used as a housekeeping gene for normalization of transcript expression (catalog no. 4319413E).

### 4.4. Statistical Analysis

All data are presented as mean ± SEM. Statistical analysis was conducted with GraphPad Prism 7.0d software (San Diego, CA, USA) using one-way ANOVA and Bonferroni’s multiple comparisons post-hoc test. Significance was determined if *p* values were < 0.05.
